# Periscope

**Published:** 1881-08

**Authors:** 


### PERISCOPE.

### North American Journal of Homœopathy, May, 1881.

Dr. Falligant writes upon dengue fever, giving a very interesting and instructive paper so far as history and diagnosis are concerned. But when he comes to the treatment, there is no homœopathy in it. Who can gather any clinical information from the prescription, in alternation, of "*Aconite* and *Arsenic* with *Ipecac*, as an intercurrent; or "*Nux V.*, *China* and *Merc. sol.*, and continue stimulants"? Is there any improvement over the poly-pharmacy of the old school in such prescriptions as the above? Is there any justification for the existence of a separate school, if these are good homœopathic prescriptions? When he boldly states, p. 546, that he gives *Aconite* and *Bryonia*, and fifteen grains of Quinine, in five grain doses," is

---

* Thirst for *hot water* and amelioration from the same, has been relieved by *Sulphur*.—Ed.

there any likelihood of convincing an old school skeptic that there "is anything in" homœopathy? Will he not say that the Quinine did the work, and the little powders had no influence?

"S. L." translates a case by Dr. Heichelheim, showing the use of *Silicea* in diseases of the bones. Principal symptoms were, in the one case: tearing in extremities; stiffness of joint in right arm; shining deep scar in the bone; thick crusty eruption in left popliteal space, *discharging acrid ichor;* left big toe stiff in joint, and covered with crusty scars; bones of toe denuded with fistulous openings, discharging ichor; offensive perspiration in axilla; somnambulism and frightful dreams. Cured in six months. Another case was inflammation of the tibia, with caries, and discharge of thin pus; itching eruption over whole body. *Silicea* (after first taking *Sulphur*). Cured in four months. The keynote of *Silicea* is *erethism, with exhaustion from malnutrition, sweat of the head, offensive sweat of feet, headstrong children.* Commenting upon some other cases, S. L. makes several comparisons, from which the following are extracted: *Sulphur* and *Psorinum* both have weakness of memory, melancholia and disinclination to work, but in *Sulphur*, "venosity" and hypochondriasis are at the root of the evil; whereas in *Psorinum* it is attributable to exhaustion. In *Sulphur* there is foolish pride and happiness, even rags appearing beautiful. The *Psorinum* patient is dull and stupid, or full of evil forebodings. Both have congestive headaches and dizziness, less marked in *Psorinum. Sulphur* has aggravation after meals, and after sleep, and amelioration when sitting, or when lying with the head high. The *Psorinum* patient feels better after eating and washing; headache and rotary vertigo are worse in mornings. *Psorinum* has aversion to having head uncovered, even in hot weather. *Sulphur* worse from heat of bed, from rest; better from motion. *Psorinum* excretions are distinguished by fetor and acridity, thus: offensive otorrhœa; offensive rhagades; nauseating discharge from nose; fœtid flatus, relieving colicky pains; fluid stools, smelling like rotten eggs or carrion; leucorrhœa in large lumps, and of fœtid odor. In *Sulphur*, fœtor takes secondary character and discharges, even if chronic or catarrhal. *Psorinum,* coughs for a long time before expectorating, from debility and exhaustion; chest symptoms better from rest, and when lying down. *Sulphur*, expectoration fails to bring relief, and the weakness in chest feels worse when lying down and talking. *Sulphur*, voluptuous tingling and itching of skin, with burning and soreness after scratching; itching worse in warm bed. *Psorinum*, the skin is dirty, greasy looking, at times itching; pustules without itching; the body has a filthy smell, even after a bath. *Sulphur*, patient imagines effluvia arising from the body which disgust him, though nobody else can detect any foul odor.

From *Hygea* is translated a case of headache *at vertex*, so severe that the whole brain seemed as if *it would be pressed assunder*. *Hyper. perfol.* cured in an hour.

In another case, where there was an accompaniment of painful, irregular menses, the same symptoms indicated the same remedy, which greatly relieved.

Some cases of whooping-congh were not relieved by the usual and apparently indicated remedies; a closer examination showed enlarged abdomen and picking at nostrils. CINA caused discharge of ascarides and cure of the cough.

Of HYPERICUM, "S. L." remarks: "It *is the arnica of the nervous system*." Excruciating pains from *laceration of the nerves*. Asphyxia after a fall; jerking, shooting pains. After a fall upon the occiput, sensation as if being lifted up in the air. Fracture of skull, bone splintered. Convulsions from blows upon the head. Epileptiform spasm after hard knocks. Prevents lock-jaw from wounds in soles of feet, palms of hands or fingers. Headache, *throbbing in vertex; the brain seems compressed.*

An unmarried woman became troubled with melancholy; indifference; aversion to work; would sit still for several hours, with eyes fixed; wept bitterly; avoided company of even her best friends; despair of recovery; suicidal ideas; thinking only of death; trembling; irritable and forgetful; sleepless at night until morning; would like to sleep all day. *Psorinum* 6th, frequently repeated caused complete cure.

Another woman had stubborn, dry cough; flying stitches in chest; dyspnœa during motion or when ascending. *Psorinum* 6th relieved immediately, and caused an eruption like itch all over the body, but mostly on chest; itching at night in bed. The medicine was stopped, and the eruption disappeared. Complete cure of all symptoms in eight weeks.

A baker had racking night cough, with copious expectoration of various colors; sometimes cheesy yellowish lumps, of an offensive odor when crushed. Every two weeks coughed up a tube-like concretion half an inch long, of greenish color and bad odor. It comes up, when there are paroxysms of cough, with anguish and suffocation. Relief follows. *Psorinum* 6th followed by *Sulph.* 1st, and then *Hepar* 1st caused complete cure.

*Sulphuric Acid* is indicated for the following symptoms: Appetite good, but food is soon vomited up; vomiting of mucus; everything vomited is sour; vomiting is perfectly easy, without nausea or faintness; whilst in company, feels a desire to vomit; leaves the room, vomits, and returns to her friends; loud eructations becoming painful; violent contractive pain in epigastrium, *temporarily relieved by port-wine or food easy of digestion;* coldness and relaxed feeling of stomach; water causes coldness of stomach, unless mixed with alcoholic liquor; desire for brandy; after eating, pain in stomch and vomiting of food; *sour vomiting.*

*Robinia pseudo-acacia* has excessive acidity of stomach; vomiting of intensely sour fluid, setting the teeth on edge; frequent eructations of sour fluids. Steady dull, heavy aching distress of stomach and sensation of soreness when moving and upon pressure. Sensation as if stomach were full of hot water, with nausea oppression and debility.

*Oxalic Acid.*—Burning pain in stomach and throat; burning pain in small spots in abdomen; slightest touch causes excruciating pain.

*Mur. Ac.*—*Putrid* eructations; gulping up of contents of stomach into the œsophagus; empty sensation in stomach, extending through whole abdomen, but no hunger.

*Nitric Acid.*—Liver and spleen enormously enlarged and deranged; several symptoms better from lying down and from carriage riding.

*Lactic Acid.*—*Eructations of hot acrid fluid,* which burns from stomach to throat. Eructations of burning hot gas from stomach, causing a profuse secretion of tenacious mucus, which must be constantly hawked up.

A young man about an hour after every meal, was obliged to vomit bitter or sour matters; loud talking or singing caused vomiting; tension over stomach worse from pressure; trouble caused probably by drugging. *Nux V.* cured.

A case of gastralgia; almost constant pain, much aggravated from binding anything tight around the wrist; sensation of contraction in stomach; tedious, dry spasmodic cough, increasing the gastralgia; cough worse from talking, running, emotions, etc. *Conium* tincture cured.

Chronic diarrhœa in a child; painless, watery or mucous stools; pale bloated face; skin cool; indentation of skin remaining after pressure of the finger; weakness from long continuance of the diarrhœa; *China* failed; *Ferrum* in massive doses cured.

An article upon " The Centre of Speech and Homœopathy by Dr. Mossa is translated from *A. H. Z.*, 4–6, 1881. We gather the following indications for aphasia.

*Bell.*—Stammering, weakness of organs of speech with full consciousness; speech difficult, whining, stammers like one intoxicated; temporary speechlessness; cannot utter a sound; low soft speech, with headache close above orbits, preventing the opening of the eyes.

*Hyos.*—Patient hears everything, but can answer only by signs and motions; tries to speak, but cannot utter a word; these symptoms had been caused by abuse of *Merc. sol.*, and were observed by Hahnemann.

*Stram.*—Constant murmuring, stammering; speech difficult and unintelligible; speaks abruptly in a raised voice—voice of higher pitch; cannot utter a sensible word, and gets angry; thinks long before being able to speak, and then it is a mere stammering; trembling of tongue when protruded.

*Cannab. sat.*—Misses words in speaking; words in torrents or else faltering in speech; repeats same word several times.

*Cannab. Ind.*—He begins a sentence but cannot finish it, because he forgets what he wants to say or write.

*Caust.*—Speech lisping, mouth drawn to right side; paralysis of right arm.

*Lachesis.*—Loss of memory; mistakes in writing; heaviness of tongue.

*Zincum.*—Repeats every question asked him in a singing tone.

Loss of memory.—For letters, *Lyc.;* for proper names, *Sulph.*, *Anac.*, *Crocus*, *Guajac.*, *Olean.*, *Puls.*, *Rhus.;* for thoughts, *Nat. mur.;* for what has been heard, *Hyos.*, *Lach.;* for what has been read, *Guajac.*, *Helleb.*, *Acid phos.*, *Staph.;* for persons, *Crocus;* for spelling, *Lach.;* for words, *Baryta*, *Lyc.* There are many other indications, but we have not space for them.

### American Observer, April.

Dr. Pope gives a review of the provings of *Kali Bichrom.*, with some clinical cases cured by its use. Among the latter may be mentioned: *Constant discharge of matter, thick and yellow, from left nostril, and having fetid smell; pain in muscles of left side of*

*neck to one small spot in side of head, brought on and made worse by blowing the nose; severe smarting pain in left nostril, extending to molar bone below the eye; a tumor of the nose, highly vascular, and filling whole cavity of right nostril with severe pain; syphilitic nodes on shin, tender to touch, with gnawing scraping pain.* It is noticeable that two important characteristics of *Kali Bichrom.* are not mentioned; they are: (1) *headache, preceded by blindness; the blindness gets better as the headache increases;* (2) *the gastric symptoms alternate with the rheumatic symptoms.* It is worthy of remark that Dr. Pope, who is usually considered as a champion of the "liberal" or eclectic sect, commits himself to the following square homœopathic sentiment: "Because a drug gives rise to a state similar to bronchitis, it does not follow that it will cure all cases of that disease. It is only that kind of bronchitis—that particular attack where the symptoms are like those a given drug will produce—and the nearer like, the better—that you can expect to cure with that drug. It is from a want of recognition of this fact that so many failures occur, in endeavoring to put the homœopathic theory into practice," There is also, in this number, a report of a case of puerperal convulsions horribly maltreated. It is followed by some comments by the editor, which are severe and just.

*Homœopathic World*, April and May. Dr. Burnett gives some cases of cough cured with *Aralia Racemosa.* The indications are: *Cough coming on at night when first lying down accompanied by asthma. Cough at night in bed after having slept an hour or two; occurring before midnight. It wakens the patient.*

Dr. Berridge gives his impressions of "Homœopathy in America." The May number opens with the ludicrous details of Lord Beaconsfield's medical treatment, in which Dr. Kidd proved to be "the man Friday" to the regulars. An interesting case of hemorrhage from extraction of teeth, with oozing of blood from gums, vomiting of blood, and flowing of blood from the bowels, were all cured by *Phos.* after styptics had failed. The case appears to have been treated by an Allopathist who thus innocently testifies to the truth of homœopathy. The *World* dares the *Lancet* to publish the case.

Dr. Berridge gives an exceedingly interesting case of hydrophobia which was wonderfully relieved by Hydrophobinum, C. M. (Swan). Although the patient died, yet the sufferings were so much relieved that it may be considered a case of true euthanasia.

*New England Medical Gazette*, April. In the proceedings of the "Worcester County Homœopathic Medical Society," is reported a case of parenchymatous nephritis characterized by albuminuria, œdema of face and hands, and morning diarrhœa thin and yellow, on rising. *Apis* 2 M cured. Also, prolapsus uteri of eighteen years' standing, with sensitiveness to cold air, dark-yellow complexion, despondency and retiring disposition, empty feeling in stomach, greenish-yellow leucorrhœa. Cured by *Sepia* 30 in four months.

